# Evaluating Dog Preference Between Artificial and Natural Turf Grasses

**DOI:** 10.3390/ani16132090

**Published:** 2026-07-06

**Authors:** Arieli D. Da Fonseca, Nathaniel J. Hall, Joseph R. Young, Edgar O. Aviles-Rosa

**Affiliations:** 1 Canine Olfaction Research and Education Laboratory, Department of Animal and Food Sciences, Texas Tech University, Lubbock, TX 79415, USA; adafonse@ttu.edu (A.D.D.F.); edgar.aviles-rosa@ttu.edu (E.O.A.-R.); 2 Department of Plant and Soil Science, Texas Tech University, Lubbock, TX 79409, USA; joey.young@ttu.edu

**Keywords:** dog welfare, canine behavior, dog parks, surface preference

## Abstract

Dog parks are widely used recreational spaces for human–dog interaction, yet there is little empirical data about how surface materials influence dogs’ behavior and welfare. This study evaluated dogs’ behavior on three surfaces commonly used in outdoor dog recreation areas. Ten dogs (N = 10) participated in ten structured play sessions in an experimental area with unrestricted access to all surfaces. The testing area consisted of a 12.2 m^2^ playground divided into nine plots of equal size. Each plot was randomly assigned a surface material (i.e., natural grass, stabilized grass, or artificial turf) in a 3 × 3 block design. Environmental and surface temperatures were recorded in each session. Dog behavior was recorded during a pre- and post-play period and measured using a 10 s scan sampling technique. In addition, surface characteristic measures were collected throughout the study to evaluate differences in their tolerance to weather conditions and usage. Artificial turf consistently reached a higher temperature (25.2 °C; 95% CI: 24.5–25.8 °C) than natural (19.4 °C; 95% CI: 18.7–20.1 °C) and stabilized (20.0 °C; 95% CI: 19.3–20.6 °C). In this study, the artificial turf reached temperatures as high as 63.8 °C while, under the same environmental conditions, the surface temperature of both natural turfgrass treatments remained below 40 °C. During the pre-play period, dogs showed more active than passive behaviors on the stabilized surface (35.03%; 95% CI: 30.58–39.80%) compared to the natural (27.86%; 95% CI: 23.94–32.10%) and artificial turf (23.31%; 95% CI: 19.81–27.20%). During the post-play period, activity levels decreased across all surfaces, while the occurrence of passive behaviors increased and was observed more frequently on the natural turfgrass surfaces (27.30% on stabilized and 15.52% on natural) than on artificial turf (2.41%). Artificial turf was less affected by dog traffic and seasonal changes; however, its surface was harder than both natural turfgrass treatments. The addition of the stabilizing grid failed to reduce soil compaction as anticipated. Overall, dogs spent more time on both natural turfgrass surfaces than artificial turf. However, a potential confounding effect of location could have influenced dog behavior. Nonetheless, our findings show that dog owners should be cautious when using artificial turf areas when environmental temperatures are above 25 °C when the turf temperature is above the safety threshold for burn injuries. These findings highlight the importance of carefully selecting surface materials for outdoor dog spaces to ensure dogs’ safety and comfort.

## Simple Summary

Dog parks are spaces where dogs play and interact, but little research has been done to understand how surface type may affect dogs’ comfort, behavior, and safety. In this study, we compared three common surface types: natural grass, stabilized grass, and artificial turf. We found that artificial turf became much hotter than the other surfaces, which may increase the risk of discomfort and injury. Hence, dog owners should be cautious when their dogs are using an area with artificial turf when environmental temperatures exceed 25 °C to prevent injuries. Our results showed that dogs spent significantly more time on natural surfaces, especially when resting. Although artificial turf remained visually more durable over time, it was also harder and less favorable from a thermal and surface hardness perspective. Although more durable, artificial turf was less utilized by dogs and its hardness and high temperature could result in injuries and discomfort. These findings suggest that the use of natural surfaces in dog parks is important to promote dog welfare and reduce potential injury and discomfort associated with artificial turf.

## 1. Introduction

There is an increasing demand for dog-friendly spaces in urban areas across the United States [1,2,3]. As a result, dog parks are one of the fastest-growing types of outdoor recreational spaces in cities. These parks offer dogs off-leash opportunities to run and play, as well as to safely engage in social behaviors with other dogs and people [2]. Access to such environments is important for dogs’ physical and emotional well-being, as opportunities for social play help reduce stress and support cognitive function [3,4]. In addition, dog parks help bring communities together and increase social connections between neighbors by offering shared public spaces where owners regularly interact and build relationships [1,3,4,5].

When building a dog park, several aspects need to be considered, including location, fencing, water availability for dogs and humans, and the type of surface material to be used. All these factors can affect a park’s usability and safety and dogs’ comfort, and could have an impact on dog behavior and welfare. Decisions about which surface materials to use are often based on construction and maintenance cost, without taking into consideration dogs’ behavior and welfare or the fact that some surfaces may be harmful or discourage dogs from running, playing, or exploring freely [2,6]. Understanding the behavioral and welfare impact that different surfaces may have is important when designing a dog recreational area because surface type can influence movement and overall comfort, and the use of different materials could even increase the risk of injury [2].

Despite its importance, very little research has been conducted to understand how surface material affects dogs’ behavior, comfort, and locomotion in recreational areas, but the existing literature on sport dogs suggests that surface material does have an impact on dog behavior, comfort, locomotion, and injuries. For instance, Hayatayi et al. [7] found that sand moisture content affected greyhound acceleration and that surface compaction was associated with higher joint impact measurements. Similarly, agility dog owners have reported better performance when competing on natural grass or dirt, indicating that surface type might affect dogs’ locomotion and biomechanics [8]. Furthermore, survey studies in agility dogs have found that there is a greater incidence of injuries when dogs are on artificial turf surfaces and that a significant number of injuries occur due to losing traction and slipping while running [8,9].

Natural turfgrass and artificial turf are two commonly used surfaces in urban dog parks [2,6]. However, to date, there is a lack of empirical research evaluating how the use of these two surfaces may affect dogs’ behavior and welfare when utilizing these areas. From a behavior and welfare standpoint, special attention should be paid to differences in surface temperature and surface compaction (hardness), since these two parameters could significantly affect dogs’ behavior and health based on existing research in sport dogs and in human athletic facilities. For instance, artificial turf tends to reach higher temperatures than natural turfgrass, particularly in hot environments [10]. Research has found that artificial turf surface temperature could be up to 30 °C higher than the temperature of natural turfgrass [11]. This can cause discomfort, thermal stress, and even injuries or burns to dogs’ paw-pads and skin [12,13]. Artificial turf surfaces have been recorded at temperatures of up to 71.8 °C under mild environmental temperatures, while under the same conditions natural turfgrass remained below 42 °C [14,15]. Veterinary research indicate that 60 s of consecutive exposure to a surface at 70 °C could cause skin damage [13]. This is of particular concern for dogs’ paw-pads, which are made of soft tissue and will be under direct contact with the surface. In addition to increasing the risk of skin burns, high surface temperatures could also increase the chances of heat stroke. Although panting is the main heat-exchange mechanism in dogs (evaporative cooling) a significant amount of heat is also dissipated via conduction [16] when dogs lay in contact with the ground. The high temperatures associated with artificial turf could reduce dogs’ ability to dissipate heat via conduction, increasing panting rate and thus the chances of heat stroke, respiratory alkalosis and hypocapnia [17].

In addition to having a significantly greater surface temperature, some artificial turf is harder than most natural grass surfaces [18]. This is because, most of the time, gravel is added underneath to promote water percolation and prevent water accumulation on the surface. Research with laboratory dogs has shown dogs have a preference for soft and padded surfaces over harder ones [19]. For instance, dogs utilized beds with fleece blankets more than beds with rubber materials and were almost never seen lying on the floor when a bed was provided [19]. This experiment shows that dogs avoid hard concrete surfaces, particularly elderly dogs, when resting and highlights the importance of providing a comfortable resting place. Harder surfaces also increase vertical pressure in dogs’ legs [20], which can increase pain and discomfort in elderly dogs and dogs with musculoskeletal conditions.

Additionally, other surface characteristics, like traction, have been found to influence dog behavior. For instance, research has found that surface traction alters animals’ gait [21] and research in agility dogs found that surface type changes the biomechanics of an animal and that different surfaces are associated with greater risk of suffering an injury [22]. For instance, research in human athletes and agility dogs found that there is a greater risk of injuries when playing or competing on artificial turf [23]. Altogether, this indicates that, relative to natural grass, artificial turf increases the risk of injury in dogs due to its hardness and slipperiness depending on the conditions. This is of special importance for elderly dogs or dogs with musculoskeletal diseases such as osteoarthritis.

Soil-stabilization grids are commonly used to reduce soil compaction and increase the durability of the grass. The main idea is that by reducing soil compaction, the grass should grow better and maintain a healthier root system, making it more resistant to traffic and environmental conditions. However, the impact that a soil-stabilization grid has on dog behavior and surface usage is still unknown. Based on the existing literature highlighting the negative consequences of soil compaction on dogs’ comfort and joint pressure, we hypothesize that the use of a soil-stabilizing grid under a natural grass surface will be beneficial because it will not only promote grass growth and climate tolerance but will also improve dogs’ comfort by reducing vertical forced pressure in their joints and legs.

Altogether, the existing research indicates that surface material affects dogs’ behavior. However, the impact of surface materials on dog behavior and dogs’ preference for different materials has not yet been evaluated outside of a sport context. Thus, the aims of this study were as follows: (1) Evaluate dogs’ usage rate for three different surfaces (natural turfgrass, natural turfgrass installed over a soil-stabilizing grid, and artificial turf) commonly used in outdoor dog areas in West Texas using a free-choice preference test. Using a free-choice test paradigm, we could evaluate dogs’ preference for the different surfaces based on the amount of time they spent interacting with them or their usage rate [24,25]. (2) Evaluate the relationship between environmental temperature and surface temperature. (3) Compare surface quality characteristics to understand changes caused by consistent dog play and evaluate differences in surface durability. We hypothesized that although the artificial turf will prove to be more durable and aesthetic, dogs will spend more time in plots with natural grass, particularly the ones with a soil-stabilizing grid, and will avoid artificial turf due to its higher temperature and hardness. Our goal is that these findings will inform future decisions about surface selection when building or remodeling outdoor dog areas, ensuring that these public spaces are not only functional but also provide an adequate surface that supports dogs’ preferences, safety, and overall well-being.

## 2. Materials and Methods

### 2.1. Ethical Statement and Animals

This study was conducted at the Texas Tech University Canine Olfaction Research and Education Lab (CORE Lab). All procedures, including animal housing and handling, were approved by the Institutional Animal Care and Use Committee (Protocol #2024-1541). Ten dogs of different breeds, ages, and sexes participated in this study (Table 1). Dogs were housed in 4.80 × 1.21 m indoor-outdoor kennels with ad libitum access to water and to the outdoor area with plastic flooring. Prior to the study, none of the dogs had prior contact or experience with the surfaces tested and all dogs underwent a veterinary examination as part of our intake exam. All dogs were part of ongoing detection dog research and participated in this study as part of the laboratory’s enrichment program, which consists of two daily 20 min enrichment sessions (e.g., a play or walk) in the morning and afternoon. All dogs were healthy and did not have any known musculoskeletal condition prior to the experiment.

The experimental playground was renovated specifically for this project, and no dog had access to it prior to the beginning of the study. Dogs were tested individually, and all sessions were conducted by one of two trained experimenters between September and December 2024. Dog testing order was randomized daily, with each dog receiving one play session per day. Testing occurred between 08:00 and 14:00 h to capture a range of environmental temperatures across the day. This allowed each dog to be tested at different times of the day with different temperatures and solar radiation. To prevent heat- or cold-related injuries, no testing was performed when environmental temperatures exceeded 32.2 °C or fell below 4.5 °C.

### 2.2. Experimental Area and Surface Treatments

**Figure 1:**
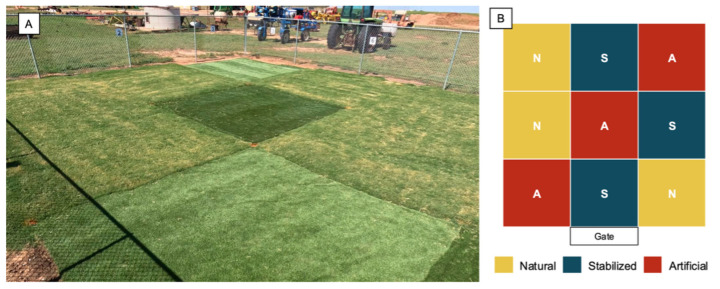
Experimental play yard layout with nine test plots. (**A**) Photograph of the play yard. (**B**) Schematic diagram showing the location of the three replicates of each surface treatment: natural (N), stabilized (S), and artificial (A). Treatment allocation was completely randomized within each block (a row in the diagram was considered a block).

The experimental area consisted of a 12.2 × 12.2 m outdoor playground (Figure 1), divided into nine plots arranged in a 3 × 3 grid. Each plot measured approximately 3.8 × 3.8 m, with a buffer of ~0.38 m maintained along the fence line. A randomized complete block design was established with three replicates of each surface treatment: (1) natural turfgrass (‘Tiftuf’ hybrid bermudagrass; *Cynodon dactylon* (L.) Pers. × *C. transvaalensis* Burtt-Davy), (2) natural turfgrass over a soil-stabilizing grid (Vevor Grass Grids, 9 ft × 17 ft × 4 in, Home Depot, Marietta, GA, USA), and (3) artificial turf (Pro-Trade K9 Olive Synthetic Turf 50 oz, SiteOne Landscape Supply, Roswell, GA, USA). The location of each plot was randomly assigned using an online randomizer, ensuring that each treatment appeared in each block of three plots (Figure 1B). These treatments are referred to as natural, stabilized, and artificial, respectively, throughout the manuscript. They were selected to represent surface materials commonly used in outdoor dog recreational areas and residential yards in West Texas. The objective of the stabilized treatment was to evaluate if soil support was effective in preventing compaction and maintaining good turf quality in a highly trafficked dog area.

**Figure 2:**
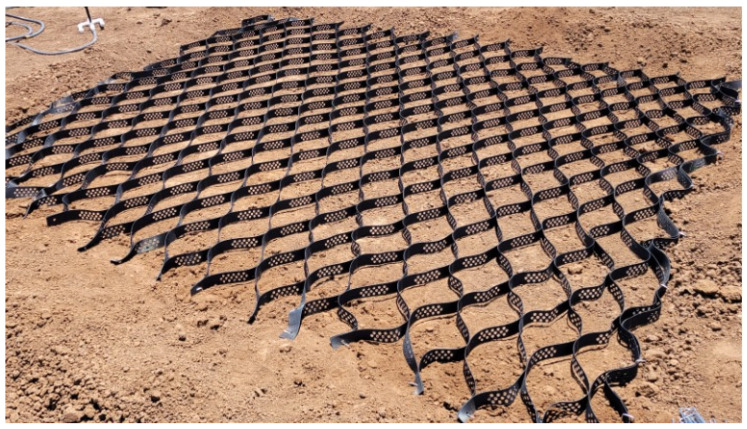
Soil-stabilizing plastic grid (stabilized) installed beneath natural turfgrass in the grid treatment plots. This should help prevent soil compaction and thus improve grass quality in high-trafficked areas.

Prior to the study, the entire yard was tilled to a depth of approximately 5 cm, and each plot was measured and delineated. Hybrid Bermudagrass sod was then laid directly on the tilled Pullman clay loam soil in the natural plots. For the stabilized surface, approximately 10 cm of soil was removed before placing the stabilizing grid, which was then covered with the same soil and topped with sod (Figure 2). This soil-stabilization system is designed to reduce soil compaction in high-traffic areas.

For the artificial plots, approximately 10 cm of soil was removed and replaced with gravel, providing a stable foundation and facilitating drainage. The artificial turf was then secured with turf stakes, with the stake heads concealed beneath the synthetic foliage. This installation method was selected to replicate common artificial turf practices [26]. An automated surface irrigation system was programmed to water the entire playground twice daily (06:00 and 18:00 h, 30 min each) to promote root establishment in the natural turfgrass plots. Once root development was confirmed by a turf specialist, supplemental irrigation was applied only when rainfall was insufficient to sustain turfgrass growth during the observation period. Testing commenced 10 weeks after sod installation to ensure proper turfgrass establishment.

### 2.3. Experimental Procedure

Each dog participated in 10 individual play sessions. During testing, dogs had unrestricted access to all nine plots. Two water bowls were positioned at opposite corners of the playground and rotated between sessions to minimize location bias, while ensuring continuous access to water and preventing heat-related injuries. Each session lasted approximately 15 min and was divided into three consecutive periods.

The first period was a 5 min habituation phase during which dogs were allowed to explore the playground freely without any human interaction (pre-play period). This was immediately followed by a 5 min interactive play period. The objective of the play period was to promote physical activity and standardize dogs’ usage of each surface to ensure dog traffic was evenly distributed among all plots and that surface quality measures (see below) were not affected by difference in usage rate. The interactive play consisted of a structured toy-fetch routine using two toys. One toy was thrown, and when the dog returned and dropped it in front of the experimenter, the second was thrown. To ensure balanced distribution of activity across plots, the experimenter alternated throws parallel and perpendicular to each block of three plots, rotating the plot from which dog started in each toss. This allowed us to balance surface usage and compare which surface better tolerated dog traffic. The final 5 min (post-play period) of the session consisted of a no-human-interaction phase, where the dog was free to display any behavior within the playground. This period allowed for observation of dogs’ resting behaviors and surface preferences when resting after a period of interactive play. The experimenter always remained inside the playground during the session to ensure dogs’ safety. To prevent the presence of the experimenter from biasing dog behavior and location during the pre- and post-play period, the experimenter was constantly walking across the playground, avoiding any specific pattern (walking in random directions), and avoided interacting with the dog (e.g., no eye contact and walking quietly). Besides the water bucket, there was no other object in the testing area during the pre-play period that could influence dogs’ behaviors. The experimenter introduced toys during the play period and the toys remained accessible to the dogs during the post-play period, even when the experimenter did not play with the dog. This was to prevent influencing dogs’ behavior, since some dogs would remain looking for the toy if the experimenter hid it. Nonetheless, dogs did not actively play with the toy after the active play period, although some dogs remained resting with the toy in their mouth.

### 2.4. Temperature Measurement

All temperature measurements were taken immediately before the start of each session. Plot temperature was not recorded within a session to prevent biasing dogs’ behavior and because minimal changes were expected within 15 min. Environmental temperature was recorded using a digital thermometer (AcuRite Indoor Humidity Monitor, Chaney Instrument Co., Lake Geneva, WI, USA). In addition, the surface temperature of each plot was measured using a handheld infrared thermometer. Because the play area had no cover or shade area, all measures were collected under normal solar radiation. Five measurements were taken per plot, one at the center and one at each corner, and the average of all measures was used for statistical analysis. These data were used to evaluate temperature variation among surface types and to examine potential relationships between environmental temperature, surface temperature, and dog behavior.

### 2.5. Behavior

All sessions were video-recorded with a GoPro Hero 11 camera mounted at 2.2 m above the ground in one corner of the playground, providing an overhead view of all nine plots. Videos were coded by a single observer using a 10 s scan sampling technique, which was validated for quantifying behavioral states and estimating activity budgets in structured settings [27,28]. A 10 s interval was selected to improve the chances of detecting infrequent behaviors, such as elimination, which are often underestimated with longer intervals [27]. Only the pre- and post-play periods were coded to assess dogs’ behavior and preferences, because dogs’ behavior during these two phases was not influenced by the experimenter or the play.

Although behavior observations were not taken during the active play, this period was important to ensure even traffic across all plots and to promote resting behaviors in the post-play period. Thus, between the pre- and post-play period, we had a total of 60 observations per session per dog, totaling 6000 observations across the study. For each observation, the experimenter recorded dog’s location (plot number) and the behavior they were performing at that instance (instantaneous scan sampling).

A detailed ethogram including operational definitions of each behavior is provided in Table 2. This allowed us to evaluate the frequency of behaviors at each plot or surface. At each observation we recorded a single mutually exclusive behavioral state from the ethogram and dog’s position within the play area. In the rare cases where a dog’s body was between plots, we recorded the dog’s location for that observation as the plot where dog’s head or more than 50% of its body was located. To facilitate analysis, all recorded behaviors were later grouped into three categories (active, passive, and elimination), which together accounted for 100% of the observations. The active category included behaviors where dogs interacted with the environment or engaged in physical activity. The passive category includes behaviors commonly observed when a dog is usually recovering after a period of high activity or seeking thermal comfort. All behavior was coded by a single observer for consistency. To evaluate intra- and inter-observer reliability, 10% of the videos were randomly selected and re-coded by the same experimenter and by a second observer. Inter-observer agreement was evaluated using Cohen’s Kappa coefficient (κ) and the percentage of agreement between observations. Results from these analyses showed high intra-observer reliability (κ = 0.87), with an overall agreement of 93.8% (*n* = 600 observations), indicating high consistency in behavioral coding by the experimenter. High inter-observer agreement (κ = 0.87) was also found with the second observer. Overall, 87.17% of the second observer’s observations were scored as identical to the main observer. This indicates the high replicability of the observations between the two different observers.

### 2.6. Surface Assessment

Turfgrass assessments were conducted prior to introducing dogs to the play area and subsequently at two-week intervals throughout the study to evaluate surface tolerance to dog traffic. Visual turfgrass quality for natural and artificial turf surfaces was rated on a 1–9 scale by a turf management expert observer, with 9 indicating a dense, dark green, and uniform canopy and 1 indicating minimal or no turfgrass coverage. Objective measures of turfgrass color and cover were obtained using a handheld normalized difference vegetation index (NDVI) meter (FieldScout TCM 500 NDVI Color Meter, Spectrum Technologies, Aurora, IL, USA) and digital image analysis from lightbox imagery [35]. NDVI values represent the mean of 15 measurements collected across each experimental unit, while digital image analysis was conducted on five arbitrary images per unit. Images were analyzed for percent green cover using TurfAnalyzer (v1.0.4) software (TurfAnalyzer, Green Research Services LLC, Fayetteville, AR, USA) [36]. Surface hardness was measured using a Clegg Soil Impact Tester (PNCLEGG-S-2.25-A, Turf-Tec International, Tallahassee, FL, USA), with values reported as the mean of 10 drops per experimental unit.

### 2.7. Statistical Analysis

For statistical analysis, each recorded behavior was classified into one of three mutually exclusive categories—active, passive, or elimination—following approaches previously used to classify canine behavioral states [33,37]. Thus, we calculated the proportion of time each dog showed active, passive, or elimination behavior on each plot during a session (Table 2). The sum of all behavior categories at each plot accounted for 100% of the observations within a session. Because the effect of plot location was confounded with treatment (e.g., each plot contained only one treatment), we could not include the effect of plot and treatment simultaneously in the model. Thus, to evaluate the main effect of surface type, we summed together each behavior category by surface to obtain the percentage of time dogs spent on each surface (sum of all plots with the same surface) and behavior category within each period (pre- and post-play) in a session. Behavioral data were analyzed using generalized linear mixed models (GLMMs) with a binomial distribution. The models predicted the proportion of time dogs spent in each behavioral category (active or passive) as a function of the fixed effects of surface type (natural, stabilized, and artificial), session period (pre- vs. post-play), and the interaction between surface type and period. Dog and testing session were included as a random effect to account for repeated measures and each plot temperature was included as a covariate in the model to account for differences in surface temperature. Model assumptions were evaluated by visually expecting the residual plots using the DHARMa package in R 4.4.1. Due to the low frequency of occurrence, elimination behavior was not statistically analyzed.

Because surfaces were randomly assigned to a specific plot and were not rotated to different plots within the experiment, we conducted an exploratory analysis to evaluate if dogs were exhibiting some location bias that could have affected the interpretation of the main effect of surface. For this, we used a GLMM as described above, with the only difference being that we included plot location and not surface type as the fixed effect.

A linear regression model was used to examine the relationship between environmental temperature and surface temperature, with surface type included as a categorical predictor of plot temperature. Statistical significance was declared at *p* < 0.05. When significant main or interaction effects were found, Tukey’s post hoc test was used for pairwise comparisons. Behavioral analyses and temperature linear regression were performed in RStudio 4.4.1 (R Core Team, Vienna, Austria). For the surface assessment variables (turf quality, NDVI, percent green cover, and surface hardness), data from multiple measurements within each experimental unit were averaged to obtain a single mean value per experimental unit (plot). These data were analyzed using the GLIMMIX procedure in SAS 9.4 (SAS Institute, Cary, NC, USA). The model consisted of the three surface types being evaluated as the fixed effect and replication as a random effect. Mean separations were performed using Least Square Means, where response variables demonstrated a significant difference at *p* < 0.05. The full data can be found in the Supplementary Materials.

## 3. Results

### 3.1. Surface Temperature

**Figure 3:**
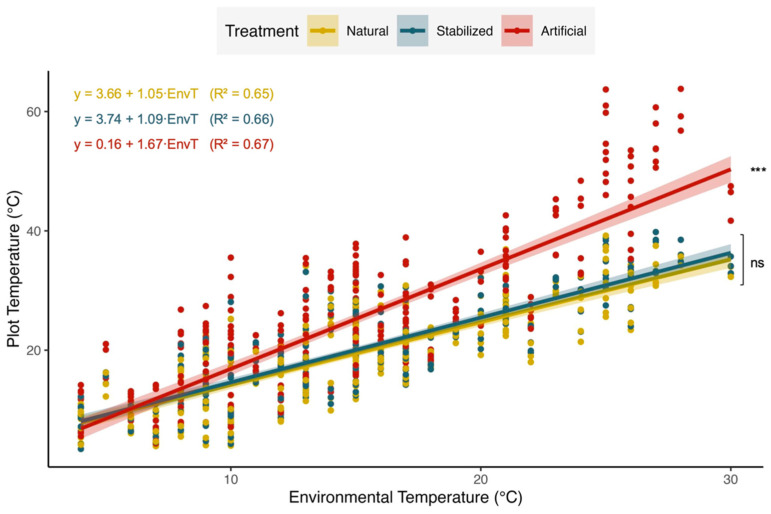
Linear regression between environmental and surface temperature. Each line represents the regression line for one of the surfaces evaluated (natural, stabilized, artificial). Shaded areas around the lines illustrate the 95% confidence interval. ns indicates no statistically significant difference and *** indicates a statistically significant difference between artificial turf and both natural turfgrass treatments.

There was a significant interaction between environmental temperature and surface type and surface temperature (F = 40.87, *p* < 0.001; Figure 3). All surfaces’ temperature increased as environmental temperature increased (F_1,880_ = 373.52, *p* < 0.001), but the rate at which the temperature increased (slope of the line) differed among treatments. The linear regression analysis showed that for every 1 °C increase in environmental temperature, surface temperature increased by 1.05, 1.09, and 1.67 °C on the natural, stabilized and artificial turf, respectively. In our study, environmental temperature ranged from 4 to 30 °C, but surface temperature varied substantially between treatments, reaching up to 63.8 °C on the artificial surface and remaining below 40 °C on the natural and stabilized surfaces at the highest environmental temperature recorded.

The mean temperature of the artificial turf (25.2 °C; 95% CI: 24.5–25.8 °C) was 5.8 °C higher than the mean temperature of the natural (19.4 °C; 95% CI: 18.7–20.1 °C) and 5.2 °C higher than the stabilized treatment (20.00 °C; 95% CI: 19.3–20.6 °C) for the duration of the study. Surface temperatures of the natural and stabilized treatments were not statistically different from each other (*p* = 0.44). Effect size tests show that, together, surface type (η^2^ = 0.17) and environmental temperature (η^2^ = 0.65) account for 82% of the variation in plot temperature.

### 3.2. Behavior

**Figure 4:**
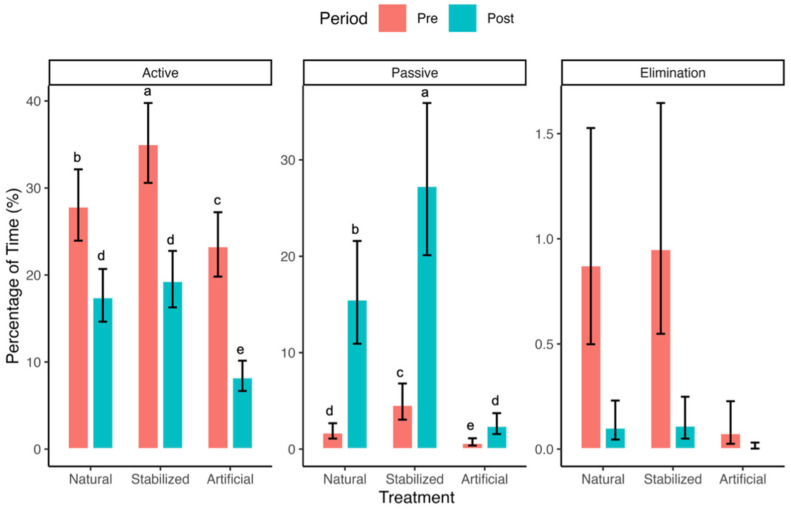
Percentage of time ± 95% confidence interval that dogs (N = 10) engaged in active, passive, and elimination behaviors across surface types and periods (pre- vs. post-). The y-axis for elimination behavior is shown on a different scale due to its low frequency. Different superscripts indicate statistical differences due to surface period interaction within each behavioral category (Tukey-adjusted, *p* < 0.05). Elimination behavior is shown descriptively due to the low frequency of events and model convergence limitations.

A significant interaction between surface type and period was found for both active (χ^2^ = 37.72, *p* < 0.001) and passive behaviors (χ^2^ = 14.81, *p* < 0.001) (Figure 4). The inclusion of surface temperature as a covariate in the model was not statistically significant for active behaviors (χ^2^ = 0.06, *p* = 0.81) but was statistically significant for passive behaviors (χ^2^ = 8.82, *p* = 0.003), indicating that surface temperature will affect dog resting behaviors but not active behaviors.

Overall, dogs showed more active behaviors during the pre-play period than the post-play period. During the pre-play period, dogs showed more active behaviors on the stabilized surface (35.03%; 95% CI: 30.58–39.80%) compared to the natural (27.86%; 95% CI: 23.94–32.10%; *p* < 0.001) and artificial (23.31%; 95% CI: 19.81–27.20%; *p* < 0.001) surfaces. During the post-play period, dogs showed a significant reduction in active behaviors across all surfaces (period effect: χ^2^ = 91.63, *p* < 0.001), with the lowest values observed on the artificial (8.25%; 95% CI: 6.68–10.20%) compared with natural (17.46%; 95% CI: 14.63–20.70%; *p* < 0.001) and stabilized (19.31%; 95% CI: 16.27–22.80%; *p* < 0.001). Active behaviors during the post-play period on the natural and stabilized surface did not differ from each other (*p* = 0.14).

As expected, dogs showed a low frequency of passive behaviors during the pre-play period, but the percentage of time spent in passive behavior differed among surfaces. Dogs spent more time sitting or lying on the stabilized surface (4.58%; 95% CI: 3.05–6.81%) compared with the natural (1.71%; 95% CI: 1.09–2.68%; *p* < 0.001) and artificial (0.63%; 95% CI: 0.36–1.12%; *p* < 0.001) surfaces during the pre-play period. Passive behaviors significantly increased across all surfaces during the post-play period, (χ^2^ = 337.84, *p* < 0.001) as expected since, after a period of play, dogs will tend to rest. Dogs spent most of their time resting on the stabilized surface (27.30%; 95% CI: 20.11–35.91%), followed by the natural (15.52%; 95% CI: 10.92–21.59%), with very little passive behavior observed on the artificial surface (2.41%; 95% CI: 1.56–3.72%).

Elimination behavior was infrequent, accounting for less than 1% of all observations. Most elimination events occurred during the pre-play period and were more common on the natural and stabilized surfaces than on the artificial surface. Due to the extremely low frequency of elimination events, statistical inference for this behavior was limited, and results are presented descriptively. Nonetheless, our results indicate that dogs almost exclusively urinated and defecated on natural grass surfaces, with fewer than 10% of all urination events occurring on artificial turf.

### 3.3. Potential Spatial Plot Bias

**Figure 5:**
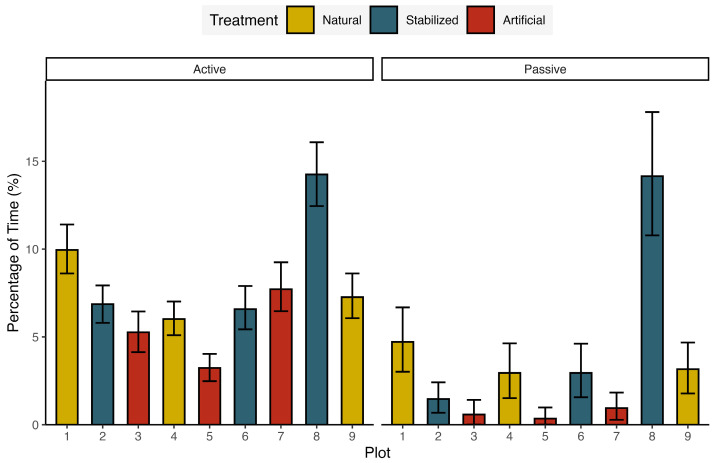
Mean percentage of time ± 95% confidence interval that dogs (N = 10) spent in each plot across the play yard for active and passive behaviors.

Figure 5 shows the percentage of time dogs spent in each plot across the play yard for active and passive behaviors. The main effect of plot location was statistically significant (*p* < 0.001). Overall, dogs spent more time in plot 8 than in any other plot, for both behavioral categories and periods. It is important to note that although this was a stabilized surface plot, because of its proximity to the gate, it could be possible that the increase in time spent in this plot could also have been influenced by its proximity to the gate. Nonetheless, due to the confounding effect of location and surface, it is not possible to separate a potential effect of proximity to the gate from the main effect of surface.

To further explore the possibility of a location bias, we conducted an exploratory analysis where we removed all the observations from plot 8 from the analysis. After removing plot 8, the interaction between surface type and period remained statistically significant for both active (χ^2^ = 35.80, *p* < 0.001) and passive behaviors (χ^2^ = 13.44, *p* < 0.01), indicating that the effect of surface type on behavior continued to differ between the pre- and post-play periods even after removing plot 8. As in our main analysis, after removing plot 8, dogs still spent more time on both natural turfgrass surfaces compared to artificial turf. However, the difference in surface area between the stabilized treatment (e.g., two plots vs. three plots in all other treatments) and the other surfaces and other statistical limitations that arose because of the removal of plot 8 from the analysis does not allow for an accurate statistical comparison between both natural forms of turfgrass. Nonetheless, this exploratory analysis confirms that, even after accounting for a possible location effect, the main effects are the same.

### 3.4. Surface Assessments

**Figure 6:**
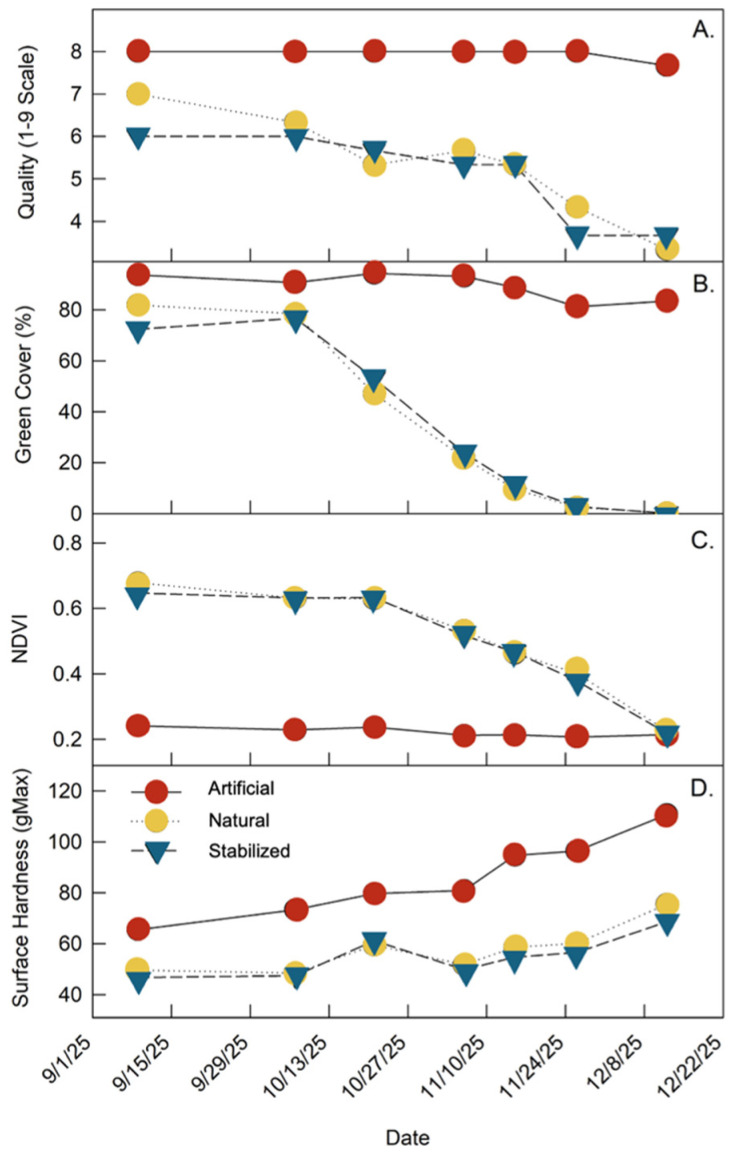
Changes in surface characteristics across sampling dates for the three treatments: artificial (red circles), natural (yellow circles), and stabilized (blue triangles). (**A**) Visual turfgrass quality (1–9 scale), (**B**) percent green cover measured through digital image analysis, (**C**) normalized difference vegetation index (NDVI), and (**D**) surface hardness measured using a Clegg Impact Tester. Values represent treatment means across the study period as dogs used the play yard and environmental conditions shifted from fall into early winter.

Across the study period, measurements showed progressive reductions in visual turfgrass quality, percent green cover, and NDVI for the natural and stabilized ‘*TifTuf*’ hybrid bermudagrass surfaces (Figure 6). These declines were more pronounced in later sampling dates as the season advanced from fall into early winter. This pattern is consistent with the expected seasonal transition of warm-season bermudagrass into dormancy as air temperatures decrease and day length shortens. In contrast, values for the artificial surface remained consistent across sampling dates.

There were significant differences among surface types for all turf-related response variables. Visual turfgrass quality and percent green cover were consistently higher for the artificial surface compared with both the natural and stabilized treatments as bermudagrass transitioned to dormancy later into fall and winter. NDVI measures light reflectance in the red and near-infrared regions, so artificial turf does not provide variable reflection as it is not a natural plant system. Surface hardness increased over time for all surface types, with the artificial turf remaining harder than both bermudagrass treatments throughout the study period.

To further explore the effect of surface hardness in dog behavior, we ran a post hoc exploratory statistical analysis including surface harness in the model. Statistical analysis showed that surface hardness was statistically significant for passive but not active behaviors. However, the main effect of treatment and the treatment period interaction remained the same as before, indicating that factors other than temperature and hardness are influencing the low usage of artificial turf. Variance inflation factor test (VIF) showed low multicollinearity between surface harness and temperature (VIF < 3).

## 4. Discussion

### 4.1. Surface Temperature

Artificial turf consistently exhibited higher temperatures than both natural turfgrass surfaces, and its temperature increased more rapidly as environmental temperature rose. The artificial turf reached temperatures exceeding 60 °C even during sessions with moderate environmental temperatures (25–28 °C), whereas both natural turfgrass treatments remained below 40 °C. These results closely align with previous research documenting extreme thermal accumulation in synthetic turf [14,15]. In contrast, the natural and stabilized surfaces demonstrated nearly identical temperature patterns, which is expected given that both rely on transpirational cooling, a physiological process in living turfgrass that dissipates heat through water loss in the canopy [38]. It is important to note that artificial turf and natural grass have different emissivity [39]. Because of this, the temperatures differences observed when using an IR thermometer are greater than the temperature differences observed with a thermometer placed over the surface [39]. For instance, Cumberbatch et al. [39] found that when measured with an IR thermometer, the difference in surface temperature between artificial turf and natural grass was 24.1 °C (like in our study) but the difference decreased to only 5.1 °C when a thermometer was placed directly over the surface. Despite this, IR thermometers are the most-used method to measure the temperature of artificial turf and grass in research.

These temperature differences have important welfare implications. High surface temperatures above 60 °C can lead to thermal discomfort, avoidance behavior, and severe skin injuries to humans and dogs’ within seconds [12,13,15]. Experimental and applied studies indicate that surface temperatures above approximately 45–50 °C are associated with an increased risk of contact-related skin injury, with burn risk increasing rapidly as temperatures rise beyond this threshold [26]. In the present study, artificial turf surfaces frequently exceeded this burn-risk limit, reaching temperatures above 60 °C under moderate environmental conditions, indicating that there could be risk of paw-pad injury with prolonged exposure. However, since we did not evaluate paw-pad injuries or thermal discomfort, future studies are needed to further evaluate the burn risk from artificial turf. Because surface temperatures are strongly influenced by solar radiation, the magnitude of heating may vary between sun-exposed and shaded areas. Thus, future studies should evaluate how a shade structure could prevent artificial turf from reaching potentially harmful temperatures.

Our findings have significant implications for pet owners. For instance, our results indicate that dog owners should evaluate the surface temperature of the artificial turf in outdoor spaces before their dog uses the area to reduce the chances of heat-related injuries and discomfort, particularly when ambient air temperatures exceed 25 °C [40]. For pet owners with artificial turf in their homes and for government agencies managing dog parks with this material, the implementation of shade or active cooling mechanisms (e.g., water irrigation, trees, tarps, etc.) may be necessary during warm conditions to ensure dogs’ safety depending on the type of turf used. Together, our findings suggest that even when artificial turf maintained better turf characteristics (e.g., color), it may be unsuitable for unrestricted canine use under high-environmental-temperature conditions, whereas natural turfgrass surfaces under the same conditions remained within a safer thermal range for use. It is important to note that we only tested a single type of artificial turf. Thus, future studies should test different types of turf to see how generalizable our results are and to identify a potential turf that can maintain lower surface temperatures.

### 4.2. Behavior

Dogs showed higher levels of both active and passive behaviors on the stabilized surface and showed the least interaction with the artificial turf compared to both natural grass surfaces. During the pre-play period, dogs showed predominantly active behavior across all surfaces and spent more time on the natural surfaces than on the artificial treatment. Following the play session, activity levels declined across all treatments, accompanied by an increase in passive behavior. This temporal shift from activity to rest is consistent with patterns reported by McGowan et al. [34], who observed elevated arousal and exploratory motivation early in interaction sessions, followed by increased resting behavior later in the session. Although McGowan et al. [34] focused on human–dog interaction under controlled conditions, the present findings suggest that similar temporal dynamics may occur in outdoor play environments.

Surface-related differences in resting behavior were also observed, particularly during the post-play period. Dogs spent substantially more time resting on the natural turfgrass surfaces than on the artificial turf surface. This indicates that the natural grass provided better thermal comfort for dogs. Previous research has demonstrated that dogs preferred softer substrates for resting, avoiding harder surfaces [19]. Based on soil compaction measurements, the artificial turf surface was harder than both natural grass treatment. Hence, the harder surface and higher temperature associated with this treatment may partially explain why dogs did not rest on it. This is an important consideration, particularly for elderly and dogs with musculoskeletal conditions, since harder surfaces will exacerbate their conditions. However, even after statistically controlling for differences in surface temperature and hardness, the data shows that dogs still spend more time on the natural grass, suggesting that other unmeasured sensory characteristics of the surface, like odor retention, traction, and texture, could also be influencing dogs’ behavior.

Based on our results, dogs urinated and defecated almost exclusively on natural grass surfaces. We hypothesize that this behavior could be due to (1) some physical limitations of the artificial turf or (2) some biological or ethological aspects of the natural grass. For instance, when urinating (particularly females) or defecating, dogs will show different squatting behaviors [41]. Although the form of elimination behavior differs by dog, it usually requires dogs to be in contact with the ground for a brief period (~0.2–2 min) of time and also requires them to put some uneven pressure on some of their legs and joints. Thus, it could be possible that dogs showed infrequent elimination on the artificial turf because its higher surface temperature and hardness made dogs avoid it. Another possibility for why dogs showed more elimination on natural grass surfaces could be due to its biological relevance. By being a natural substrate, natural grass could provide dogs with biologically relevant olfactory cues for scent-marking. Its lower temperature also makes it ideal substrate for marking, since the lower temperatures will allow for a longer retention of pheromones and other biological odors to facilitate chemical communication between conspecifics. Studies in human sporting facilities suggest that artificial turf has also a greater count of pathogenic bacteria [42] when compare to natural grass surfaces. Although, to our knowledge, this has not been explicitly investigated in dog areas, another possibility for the limited use of artificial turf could be that dogs avoided it due to its higher bacterial count. However, this is highly speculative and should be further evaluated in future research. Nonetheless, this highlights the need for proper sanitation of artificial turf areas to avoid health problems in dogs.

### 4.3. Surface Condition Dynamics

Seasonal patterns in turfgrass conditions aligned with expected physiological transitions of warm-season bermudagrass in West Texas. As day length and temperatures declined, both the natural and stabilized plots showed progressive reductions in visual quality, percent green cover, and NDVI, reflecting dormancy onset and canopy loss. Dog traffic likely accelerated these declines, particularly in the bermudagrass treatments, where mechanical wear can compound seasonal senescence. This interaction between seasonal senescence and mechanical wear highlights the importance of turfgrass selection when natural surfaces are used in high-traffic canine environments.

In this instance, the newer hybrid bermudagrass ‘*Tiftuf*’ maintained adequate surface cover, even as winter dormancy became prevalent later in the study. Both bermudagrass treatments were maintained under the same mowing management prior to the start of the study, and no mowing occurred during the experimental period, resulting in similar canopy structure between the natural and stabilized plots. Furthermore, the high-traffic area near the gate (plot 8) maintained reasonable brown turfgrass cover despite consistently higher levels of dog traffic throughout the study period. Other natural turfgrass species or bermudagrass cultivars may not be capable of handling this level of traffic and may have a higher probability of thinning and developing greater soil compaction with reduced turfgrass cover and continued dog traffic. In contrast, the artificial surface showed minimal changes across sampling dates, consistent with its non-living structure and resistance to traffic-related degradation.

Surface hardness increased over time for all treatments, but artificial turf remained consistently harder than bermudagrass surfaces. No differences in hardness were observed between stabilized and natural treatment, suggesting that the stabilizing material used did not prevent soil compaction as expected. Despite our hypothesis, our results show that the addition of a stabilizing grid had no impact on turf quality and that both natural surfaces had similar characteristics.

A post hoc exploratory statistical analysis including surface harness as a main effect in the model showed that its effect on dogs’ behavior was statistically significant. Nonetheless, even after correcting for surface hardness in the model, the main effect of surface remained highly significant, indicating that other factors like texture and smell are also influencing dog behavior. The fact that there was complete separation of the hardness between surfaces, and thus high collinearity between surface type and hardness, limits our ability to accurately evaluate the effect of hardness on dogs’ behavior. To further evaluate the main effect of surface hardness, future studies where the surface hardness of different surfaces can be experimentally manipulated should be conducted. Nonetheless, our data suggests that surface hardness does have an impact on dogs’ behavior. This mechanical stiffness may have implications for canine comfort and joint loading during locomotion, particularly over repeated exposures [20,21]. Together, these findings highlight that surface condition is both dynamic and surface-dependent, with natural turfgrass systems being sensitive to seasonal and traffic effects, whereas artificial turf maintains structural stability but may pose mechanical and thermal disadvantages for dogs.

### 4.4. Implications for Surface Selection and Dogs’ Welfare

Altogether, the thermal and behavioral findings of this research support the use of natural turfgrass over artificial alternatives in canine recreation areas. Dogs used both natural turfgrass treatments (natural and stabilized) more frequently than artificial turf during both the pre-play and post-play periods. These behavioral patterns align with prior evidence suggesting that surface properties influence dogs’ use of outdoor spaces, particularly when thermal comfort is compromised [43].

The addition of the stabilized material did not prevent soil compaction or increase tolerance to weather conditions and dog traffic as expected. Although prior research has suggested that stabilization grids can improve soil structure and reduce compaction in high-traffic environments [26], the present findings suggest that they do not inherently improve grass or soil characteristics relative to standard turfgrass installation when used in a dog area. Additional studies may be needed to determine whether other soil-stabilization systems provide long-term durability or welfare advantages under heavier traffic conditions or in multi-dog environments.

In terms of durability and maintenance, the artificial turf was the surface least affected by weather conditions and dog traffic. However, from a behavioral and welfare perspective, it exhibited the greatest limitations and risks among the surfaces tested. These findings highlight the need for caution when selecting synthetic materials for dog parks, particularly in regions prone to high temperatures.

### 4.5. Limitations and Future Directions

At face value, the behavior data may suggest a preference for the stabilized surface. However, spatial analyses revealed that one stabilized plot (plot 8), located adjacent to the gate, received disproportionately higher levels of dog activity. Prior research has found that dogs can cluster near barriers or gates, particularly during transitions or periods of reduced stimulation [44]. Thus, it could be possible that the increase in time shown in the stabilized surface could be due to the confounding effect of the gate location and not a real treatment effect. Even after removing plot 8 from the statistical analysis, our results still show that dogs spent more time on both natural turfgrass surfaces than the artificial, our inability to statistically separate a possible confounding effect of location from the treatment effect does not allow us to accurately say there was a treatment effect of the stabilizing grid. Because soil compaction, temperature, and other turf characteristic variables did not differ between both natural turfgrass treatments, we speculate that the observed difference between the two natural treatments is mostly due to proximity to the gate and not a real treatment effect. Nonetheless, even though this confounding effect does not allow us to confidently say that there was a real difference between the natural and stabilized treatments, our results still reflect a strong preference for both natural turfgrass treatments over artificial turf. Future research should carefully consider treatment placement and spatial balance to minimize external sources of bias. Rotating the different treatments across the play area would have been ideal to avoid location bias. However, due to the outdoor nature of the research and the cost and difficulty of rebuilding a new testing area, treatments could not be rotated in our research since that would have required us to remove all the surfaces and rebuild the testing area. This limited our ability to include the effect of location in the model and better assess location bias. To address this important limitation of our current study and avoid possible confounding effect of locations, futures studies should use multiple play areas where all the surfaces are randomized in different orders, include more entry and exit points within the testing areas, or design a system that would allow for randomization of the surface in different areas in an easy and cost-effective way. Additionally, these findings highlight the importance of evaluating how external factors, such as shade, fencing, and enclosure layout, may interact with surface type to influence dogs’ space use and preferences.

Our study was conducted during the fall and early winter. This limits our ability to predict the welfare risk during summer when temperatures will be higher. Nonetheless, because the artificial turf reached higher temperatures than the natural turfgrass during early fall, we expect that the thermal patterns observed during summer months may produce even stronger contrasts among surface types. It is important to note that the play yard used in this study did not contain shaded areas, and all plots were exposed to direct sunlight during the observation periods. Thus, the plot temperature collected at the beginning of a session could have been different from the temperature at the end of the session. This limitation could be avoided by collecting plot temperature multiple times within a session. However, herein we only collected at the beginning of the session to avoid influencing dogs’ behavior within a session. Future studies should evaluate dog behavior and surface temperatures across different seasons in a group setting, since the presence of other dogs might influence dogs’ behavior, when treatments are rotated in different location, and when shade is provided over the artificial turf. Furthermore, the evaluation of dog physiological markers like paw temperature, cortisol, and gait analysis are needed to be evaluated to better understand the effects of surface temperature on dogs’ behavior, welfare and health.

Another important limitation is that we only tested a single artificial turf and installation procedure. For instance, there are commercially available padded artificial turf designed to reduce surface hardness. Thus, it is important to further evaluate if our results could be generalized to other turf materials or if the cushion provided by other types of turf will change dogs’ behavior. The further testing of different artificial turf materials indoors or under complete shade is necessary to further understand the underlying factors influencing dogs’ behavior and to determine the optimal type and usage of artificial turf in dog recreational areas. Future studies should also try to separate the natural effect of season dormancy and dog traffic on natural turf qualitative measurements. Herein, it was not possible to separate each effect, and future studies with a control area with no dog traffic would provide a better insight into the effect of dog traffic on the qualitative characteristics of the grass.

Despite these different limitations, our study provides preliminary evidence that highlights the impact of different surfaces on dogs’ behavior and the need for future research in this area.

## 5. Conclusions

Our results show that dogs spent more time on both natural turfgrass surfaces when compared to artificial turf. Natural turfgrass had consistently lower temperatures and less compaction than artificial turf. Artificial turf reached temperatures as high as 63.8 °C under mild environmental conditions, while natural turfgrass never exceeded 40 °C. Thus, dog owners should be careful when utilizing areas with artificial turf when the environmental temperature exceeds 25 °C since its surface temperature could cause injuries to dogs. Together, these results suggest that natural turfgrass surfaces represent a more suitable option for canine recreation areas with no natural or artificial shade areas, as they may help to maintain lower surface temperatures. The results also highlight the need for dog owners and urban planners to incorporate irrigation and shade in areas where artificial turf is utilized under direct sunlight. Future studies should further evaluate other artificial turf materials, the impact of shade areas, and physiological markers to better understand the impact of artificial turf on dogs’ welfare.

## Figures and Tables

**Table 1:** Dog demographics.

Dog	Breed	Age (Years)	Sex/Neuter Status
Adele	Belgian Malinois	4	F (spayed)
Bessie	German Shorthair Pointer	3	F (spayed)
Boomer	Springer Spaniel	4	M (neutered)
Foxy	Mixed breed	2	F (spayed)
Iitooma	English Springer Spaniel	4	M (neutered)
Oakwood	Mixed breed	1	M (neutered)
Purdey	Labrador	3	F (spayed)
Stan	Mixed breed	1	M (neutered)
Wolfgang	Mixed breed	2	M (neutered)
Zulu	German Shorthair Pointer	6	M (neutered)

**Table 2:** Ethogram of dog behavior.

Category	Behavior	Description	Reference
Active	Locomotion	The dog moves forward within the enclosure using coordinated limb movements, resulting in a change in overall body position, regardless of speed while not sniffing the ground.	[29]
	Standing	Dog observed in an upright position, with all four paws in contact with the ground and body supported by the limbs, while not actively sniffing or moving their head from side to side to explore the environment	[29]
	Exploratory behavior	The dog investigates the environment through sniffing, looking around, or approaching objects or surfaces.	[30]
	Self-directed behaviors	The dog performs behaviors directed toward its own body, such as scratching, liking, or shaking.	[31]
	Drinking	The dog positions its mouth over or in the water bowl and performs repeated lapping motions with the tongue.	[29]
	Digging	The dog uses one or both forepaws to repeatedly scratch and displace the ground surface.	[29,32]
	Rolling	The dog rolls its body on the ground, typically exposing the abdomen, and may show body- or limb-stretching, often associated with play or comfort-seeking behavior.	[31,33]
Passive	Sitting	The dog remains stationary with its hindquarters resting on the ground and forelimbs extended for support; the base of the tail is in contact with the ground while not actively sniffing or moving their head from side to side to explore the environment.	[29,34]
	Lying	The dog is stationary in a ventral or lateral recumbent position, with the sternum, torso, or side in contact with the ground. The head may rest on the ground or be held upright.	[29,33]
Elimination	Urination	The dog adopts a squat or leg-lift posture and releases urine.	[29]
	Defecation	The dog adopts a squat posture and expels feces.	[29]

## Data Availability

The data presented in this study are available from the corresponding author upon reasonable request.

## Supplementary Materials

The following supporting information can be downloaded at: https://www.mdpi.com/article/10.3390/ani16132090/s1.
